# Magnetic nanoparticles as targeted delivery systems in oncology

**DOI:** 10.2478/v10019-011-0001-z

**Published:** 2011-01-19

**Authors:** Sara Prijic, Gregor Sersa

**Affiliations:** 1 Nanotesla Institute, Ljubljana, Slovenia; 2 Institute of Oncology Ljubljana, Department of Experimental Oncology, Ljubljana, Slovenia

**Keywords:** magnetic nanoparticles, nanotechnology, delivery systems, oncology, magnetofection, cancer therapy, magnetic targeting

## Abstract

**Background:**

Many different types of nanoparticles, magnetic nanoparticles being just a category among them, offer exciting opportunities for technologies at the interfaces between chemistry, physics and biology. Some magnetic nanoparticles have already been utilized in clinical practice as contrast enhancing agents for magnetic resonance imaging (MRI). However, their physicochemical properties are constantly being improved upon also for other biological applications, such as magnetically-guided delivery systems for different therapeutics. By exposure of magnetic nanoparticles with attached therapeutics to an external magnetic field with appropriate characteristics, they are concentrated and retained at the preferred site which enables the targeted delivery of therapeutics to the desired spot.

**Conclusions:**

The idea of binding chemotherapeutics to magnetic nanoparticles has been around for 30 years, however, no magnetic nanoparticles as delivery systems have yet been approved for clinical practice. Recently, binding of nucleic acids to magnetic nanoparticles has been demonstrated as a successful non-viral transfection method of different cell lines *in vitro*. With the optimization of this method called magnetofection, it will hopefully become another form of gene delivery for the treatment of cancer.

## Introduction

Nanotechnology is an interdisciplinary field of technological developments on the nanometer scale offering comprehensive applications also to biomedicine. Engineering particles a several tens of nanometers in diameter has opened new possibilities for targeting cells within an organism either for diagnostic or therapeutic purposes.

Magnetically-guided drug or gene targeting using magnetic nanoparticles is a promising approach for cancer chemotherapy and cancer gene therapy. The rationale behind these two treatment modalities is based on binding either chemotherapeutics or nucleic acids onto the surface of magnetic nanoparticles which are directed to and/or retained at the tumor by means of an external magnetic field. Researchers have been studying magnetically-guided drug targeting since the late 1970’s^1^, however, magnetically-guided gene targeting has emerged as rapid and efficient approach in the beginning of the new millennium.^2^ Magnetic nanoparticles have been explored predominantly in basic and translational research in the field of oncology although some of them have been already clinically approved as contrast enhancing agents for magnetic resonance imaging (MRI).^3^

## Magnetic nanoparticles

### What are nanoparticles?

To date, there is no uniform definition of a nanoparticle. According to Kreuter, a nanoparticle is a solid colloidal particle ranging in size from 1 to 1000 nm.^4^ In nanomedicine, »nano« can be applied to materials or surfaces that are intentionally altered and manipulated at nanometer scale resulting in new properties.^5^ Besides the differences in size, nanoparticles are also distinguished based on their shape and chemical composition (Table 1).

**FIGURE 1.**
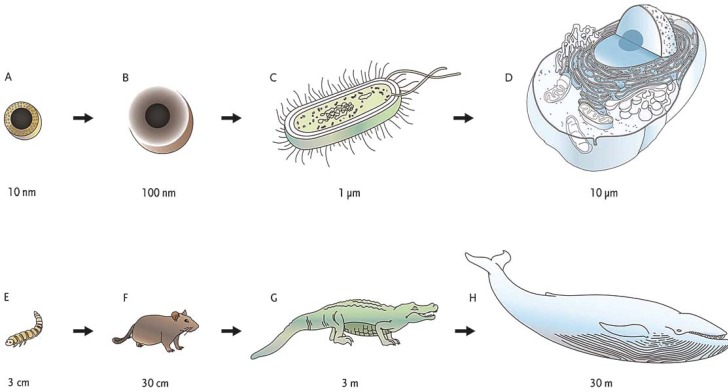
Illustrative demonstration of size comparison of a nanoparticle at the microscopic level with corresponding relations on the macroscopic level. Sizes at the microscopic level (A–D) are equivalent to the ones at the macroscopic level (E–H). Magnetic nanoparticle coated with a thin inorganic layer (A), magnetic nanoparticle coated with an organic polymer (B), prokaryotic cell (C) and eukaryotic cell (D) are in the same size relation as a mealworm (E), a rat (F), an alligator (G) and a blue whale (H), respectively.

Despite the fact that many nanoparticles measure more than 100 nm in one dimension, a novel explanation of a nanoparticle based on the following foundations has emerged. First, the majority of nanoparticles for biomedical applications are prepared as colloidal dispersions, *i.e.* homogenous chemical mixtures of two separated phases. The homogeneity of dispersed-phase particles into a continuous-phase aqueous medium is only possible if dispersed-phase particles have a diameter between 5 and 200 nm.^6^ Second, unique differences of physical properties that distinguish nanoparticles from atoms as well as from the bulk material are the most prominent below 100 nm. Hence, a nanoparticle is defined as a particle of any kind of material which has one or more dimensions equal to or smaller than 100 nm (Figure 1).^7^

### What are magnetic nanoparticles?

Nanoparticles consisting of iron, nickel and/or cobalt which exhibit magnetic properties are called magnetic nanoparticles.^8^ Elemental manganese which has a complex crystal structure and unusual magnetic properties can also display magnetic behavior after special physicochemical treatment.^9^

Briefly, the magnetic properties of a material are the reflection of magnetization which arises from magnetic moments of unpaired electrons due to their orbital motion around the nucleus of an atom and intrinsic spinning around their axes. Due to the thermal fluctuations of magnetic moments that reverse direction, some magnetic nanoparticles exhibit superparamagnetic properties which are defined as the nonappearance of magnetic behavior when the magnetic field is not present.^10^ Superparamagnetic properties are observed at sizes smaller than 15 nm for iron oxide maghemite (γ-Fe_2_O_3_).^11^ Hence, magnetic nanoparticles which are small enough, composed of iron oxide and display magnetic behavior only in the presence of a magnetic field are called superparamagnetic iron oxide nanoparticles (SPIONs). It is essential to use SPIONs in biomedical applications since the permanent magnetic behavior of magnetic particles within an organism would be redundant or even destructive when the magnetic field is removed. For example, magnetically induced deformation of endosomes containing paramagnetic nanoparticles was shown by the transmission electron microscope (TEM).^12^

Most studies discussed in this review refer to SPIONs, however not all of them. Hence, the superior term magnetic nanoparticles will be used also for SPIONs in order to make the manuscript more lucid and organized whereas the term SPIONs will be used only when emphasizing the importance of superparamagnetic behavior of the nanoparticles. The majority of magnetic nanoparticles as targeted delivery systems are chemically iron oxides (Table 1). Iron is essential to nearly all known organisms and even endogenic iron oxide nanoparticles were detected in the human hippocampus.^13^,^14^ However, at the cellular basis, iron oxide causes direct cytotoxicity due to the generation of oxygen and nitrogen-based atoms with an unpaired electron, *i.e.* reactive oxygen and nitrogen species (ROS and RNS).^15^ Therefore, magnetic nanoparticles are predominantly prepared through the use of core-shell methodology. As reviewed by Gupta and Gupta, the magnetic core of iron oxide nanoparticles is composed of magnetite (Fe_3_O_4_) and/or maghemite (γ-Fe_2_O_3_) whereas their shell surface coating can be of organic compounds, including surfactants and synthetic or natural polymers, or inorganic material, such as silica, carbon, precious metals or oxides.^16^ Synthesis of core-shell type magnetic nanoparticles is important due to the several reasons: (i) protection of the magnetic core from oxidation, (ii) protection of the surface from chemical reactions, (iii) avoidance of aggregates and agglomerates formation due to Van der Waals forces, hydrophobic effects and magnetic attractions, (iv) facility of the various therapeutics attachment and (v) amplification of the cellular uptake rate.^17^

Biocompatibility of magnetic nanoparticles depends on the type of their surface coating which can be biodegradable (*e.g.* certain polymers) or non-biodegradable (*e.g.* silica) as well as on their size. The thickness of the coating determines the total size of magnetic nanoparticles, *i.e.* coating with an inorganic material in general results in smaller particles below 100 nm whereas a polymer coating is predominantly reflected in larger particles above 100 nm.^18^,^19^ The type of the coating determines characteristics of the particle surface, such as hydrophilicity and surface charge.

For biomedical applications magnetic nanoparticles are predominantly prepared as ferrofluids, *i.e*. magnetic liquids, thus their surface charge is established by ionization of surface groups or by adsorption of charged species of a surrounding liquid medium onto the particle surface which results in a layer around the particle. The potential difference between the surrounding liquid medium and the layer around the particle is called the zeta potential. Particles with a zeta potential higher than 30 mV, either positive or negative, will repel each other, stay asunder and result in a stable ferrofluid.^6^

### Why use magnetic nanoparticles?

The use of magnetic nanoparticles as drug or gene delivery systems can contribute to the effectiveness of cancer therapy in many ways. First, an advantage of using magnetic nanoparticles over non-magnetic ones is that magnetic behavior allows monitoring and quantitative determination of their biodistribution by MRI, which facilitates optimal dosing in cancer therapy. Second, targeting of tumors by magnetic nanoparticles can overcome some supplementary hindrances in more efficient treatment of cancer, such as insufficient penetration of certain therapeutics from the bloodstream into the tumor. Third, targeting of tumors with magnetically-guided nanoparticles provides site specificity and thus selectivity of the therapy, which results in reduced side effects and lower cost of the therapy. And last, exploiting the magnetic field as the driving force represents a non-invasive therapeutic approach.^10^

### How to exploit magnetic nanoparticles?

The basic principle of using magnetic nanoparticles for targeting in oncology is to increase antitumor efficacy while at the same time reduce undesired systemic side effects towards normal tissues by (i) passive targeting, (ii) active targeting and/or (iii) targeting with an external magnetic field. Passive and active targeting can be achieved irrespective of nanoparticles possessing magnetic behavior. Passive targeting is referred to the extravasation of nanoparticles from the blood-stream into the tumor due to disorganized and leaky tumor vasculature.^20^ Active targeting is related to appropriate ligands, predominantly monoclonal antibodies, their fragments, inhibitors of tyrosine kinase domains and most recently aptamers, which actively target tumor markers and are attached to nanoparticles.^21^ Ligands can target extracellular matrix, surface receptors on endothelial cells of tumor vessels or pericytes and tumor cell surface receptors.^22^ On the other hand, targeting by means of an external magnetic field can only be possible in the case of magnetic nanoparticles. Combining active targeting and targeting with a magnetic field provides double-targeting where the magnetic field represents an initial targeting vector that concentrates magnetic nanoparticles in the tumor followed by second level active targeting by means of ligands, bound onto the surface of magnetic nanoparticles that are specific for tumor cells.

Magnetic nanoparticles are in most cases manipulated by a magnetic field which is generated by high-field, high-gradient, rare earth magnets, such as neodymium iron boron (Nd-Fe-B) magnets. Nd-Fe-B magnets are the strongest type of permanent magnets which have been commercialized not earlier than 1986.^23^ The basic principle of magnetically-guided targeting is to place a magnet over a targeted site, *i.e.* a tumor, in order to *in situ* concentrate and/or retain magnetic nanoparticles.

If targeting with an external magnetic field is in conjunction with bare magnetic nanoparticles with no attached therapeutics, such a kind of cancer therapy relies on intentional obstruction of tumor vessels. Cancer therapy aiming to obstruct tumor vessels with magnetically guided microparticles composed of carbonyl iron was performed in the early 1970s. Unfortunately, the material displayed low *in vivo* stability as well as a low ability to be guided by the magnetic field.^24^ More than two decades later, magnetic nanoparticles were intravenously administrated to mice prior to applying the magnetic field with a flux density of 200–500 mT for 20 min above the subcutaneously transplanted colon carcinomas or hypernephromas. This caused complete and permanent tumor remissions due to tumor blood vessels obstruction.^25^ Compared to active targeting with antibody-bound magnetic nanoparticles, targeting with bare magnetic particles by sufficiently long exposure (6 h) to the magnetic field with a flux density of only 2.5 mT resulted in comparable retention at the targeted site, in this case lungs and heart.^26^

## Pathways of magnetic nanoparticles within an organism

Efficient internalization of magnetic nanoparticles into targeted cells and subsequent therapy outcome are limited by several factors, such as (i) cytotoxicity, (ii) nanoparticle aggregation due to increased surface/volume ratio and (iii) short plasma half-life due to their elimination from the bloodstream by phagocytic cells. In general, biocompatibility of magnetic nanoparticles mainly depends on their physicochemical properties whereas the route of administration and characteristics of an applied magnetic field also affect cellular uptake and biodistribution.^27^,^28^

### Cellular pathways

It is known that different therapeutics get in contact with cells mainly by Brownian motion during a given incubation time. Hence, the crucial limitation in achieving more efficient delivery of therapeutics to the cells is the lack of contact between the delivery system and cellular surface. The contact between the therapeutics and cellular surface can be increased by exploiting the gravitational force^29^ as well as magnetic force.

#### Sedimentation

Manipulating the magnetic force *in vitro* leads to accelerated sedimentation of magnetic nanoparticles onto the cellular surface and does not directly affect their internalization.^30^,^31^ Magnetic nanoparticles exposed to Nd-Fe-B magnets with the remanence of approximately 1 T, *i.e.* the strength of the magnetic field at the core of the magnet, were detected *in vitro* onto the cellular surface within a few minutes.^32^

#### Internalization

Once the magnetic nanoparticles are located onto the cellular surface, fast internalization begins. Results obtained by mechanics modeling demonstrate that particles in the size range of tens to hundreds of nanometers can enter cells even in the absence of clathrin or caveolin-mediated endocytosis.^33^ However, the majority of experimental studies concluded that internalization of magnetic nanoparticles was mediated through endocytosis, beginning with the invagination of the plasma membrane at either clathrin-coated pits or caveolaes.^18^,^31^,^34^–^36^ The extent of involvement of clathrindependent and caveolae-mediated endocytosis seems to be cell dependent.^31^

Irrespective of nanoparticles possessing magnetic behavior, authors of studies have reported about more efficient membrane crossing and cellular uptake of smaller particles in comparison to larger ones, *e.g.* 10–20 nm *vs.* 1000 nm and 70 nm *vs.* 200 nm.^37^,^38^

**FIGURE 2.**
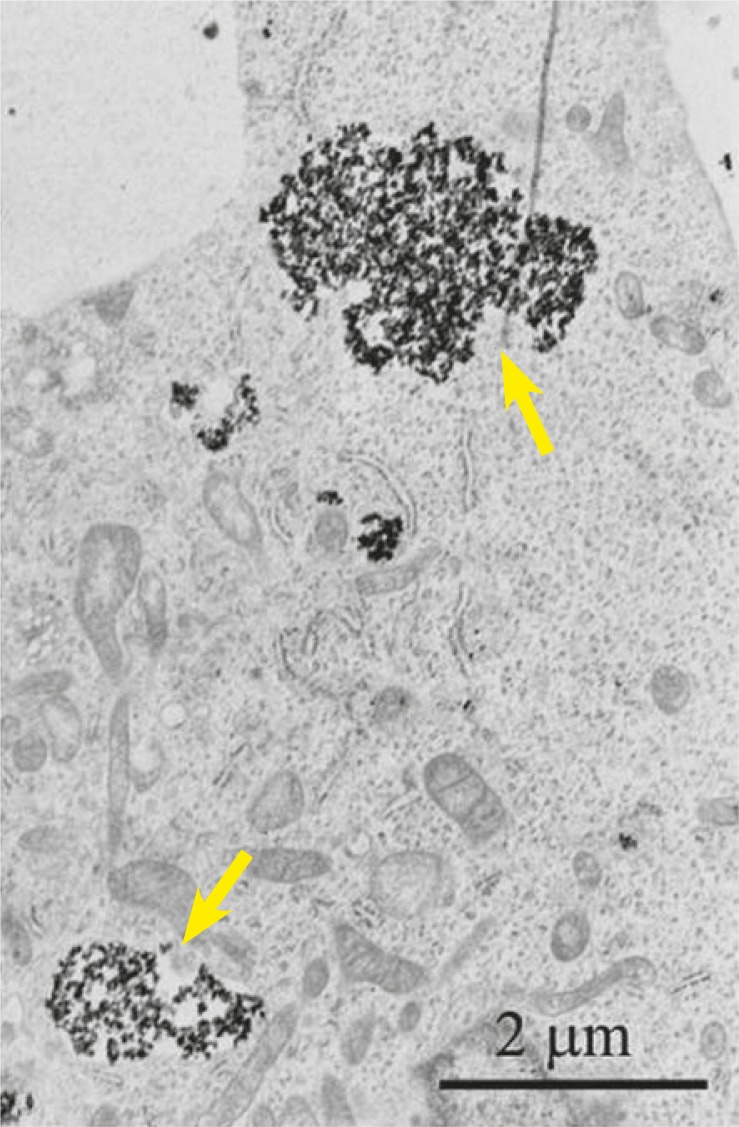
Transmission electron micrograph of human melanoma SK-MEL-28 cell, taken 4 h after the cell has been exposed to 100 μg SPIONs/ml. Arrows indicate enlarged endosomes with high accumulation of SPIONs.

Malignant cells are more prone to internalization of magnetic nanoparticles than normal cells (Figure 2).^18^,^35^,^39^,^40^ The reason is that malignant cells possess a higher endocytotic potential than normal cells due to their enhanced requirement for nutrients in virtue of their high metabolic activity and proliferation rate.^41^ This makes magnetic nanoparticles especially suitable for delivery of anticancer therapeutics into tumor cells.

#### Cellular trafficking

Once internalized, magnetic nanoparticles with attached therapeutics remain within the maturing endosomes until they fuse with lysosomes where they are exposed to digestive enzymes. Linkage between a magnetic nanoparticle and a therapeutic has to overcome degradation within body fluids but has to achieve fast and simple cleavage once magnetic nanoparticles are internalized into cells. Various molecules can be linked to magnetic nanoparticles in order to release their cargo from the endocytotic-degradative pathway.

In the case of gene delivery, endosomal escape of nucleic acids is in most cases achieved by the proton sponge effect of endosomolytic polymers, such as polyethylenimine (PEI).^31^,^42^–^44^ Due to the large number of amino groups PEI possesses a high buffering capacity which in the acidic environment of the endolysosomes induces proton entry and accumulation, followed by passive chloride influx leading to osmotic swelling of the endolysosomes. Endolysosomes burst releasing their content into the cytoplasm.^45^ Irrespective of the applied magnetic field, binding of PEI to the surface of magnetic nanoparticles increased transfection efficiency of anti-GFP siRNA in stably transduced cervical cancer HeLa cells with GFP for 20% at siRNA concentrations as low as 8 nM.^42^ Moreover, under magnetic field guidance, addition of free PEI to already PEI-modified magnetic nanoparticles resulted in an approximately 8-fold increase in transfection efficiency of the *luciferase* reporter gene to Swiss albino mouse fibroblasts (NIH 3T3) in comparison to transfection using just PEI-modified magnetic nanoparticles.^42^

However, as PEI is vastly cytotoxic^44^,^46^, also other molecules are attached to magnetic nanoparticles, such as fusogenic peptides and cell-penetrating peptides (CPPs).^28^,^47^,^48^ Fusogenic peptides, *e.g.* INF-7, are able to form membrane channels in response to low pH which leads to the disruption of an endosome.^49^,^50^ CPPs, *e.g.* Tat peptides, are a family of proteins containing short cationic or amphiphatic polypeptide sequences, termed the protein transduction domain that have the ability to cross cellular membranes while carrying macromolecules.^51^,^52^

#### Penetration into the nucleus

The nuclear membrane allows passive transport of substances below 50 kDa whereas other substances can only enter the nucleus by active transport through nuclear pore complex (NPC). NPC consists of importins, heterodimeric proteins of α and β subunits, which bind to a specific recognition sequence called the nuclear localization signals (NLSs) of the importing substance. NLSs consist of arginine and lysine sequences which help in the transportation of the substance from the cytoplasm to the nucleus.^53^ In order to avoid the use of the intrinsic machinery of the viruses to enter the nucleus and to enhance transfection efficacy, synthetic NLSs have been produced and bound to magnetic nanoparticles. For example, magnetic nanoparticles successfully entered the nucleus of HeLa cells only when modified with NLSs peptide.^54^

### Biodistribution within an organism

As reviewed by Soenen and Cuyper, biodistribution of magnetic nanoparticles depends on their physicochemical properties, such as size, hydrophilicity and surface charge.^15^ Irrespective of nanoparticles possessing magnetic behavior, with increasing their surface charge and decreasing hydrophilicity, the capacity of plasma protein absorption increases which leads to their recognition by phagocytic cells.^55^ By increasing the size, renal clearance is omitted, however, nanoparticle recognition by phagocytic cells increases which results in their accumulation in the liver, spleen and lymph nodes.^56^

**FIGURE 3.**
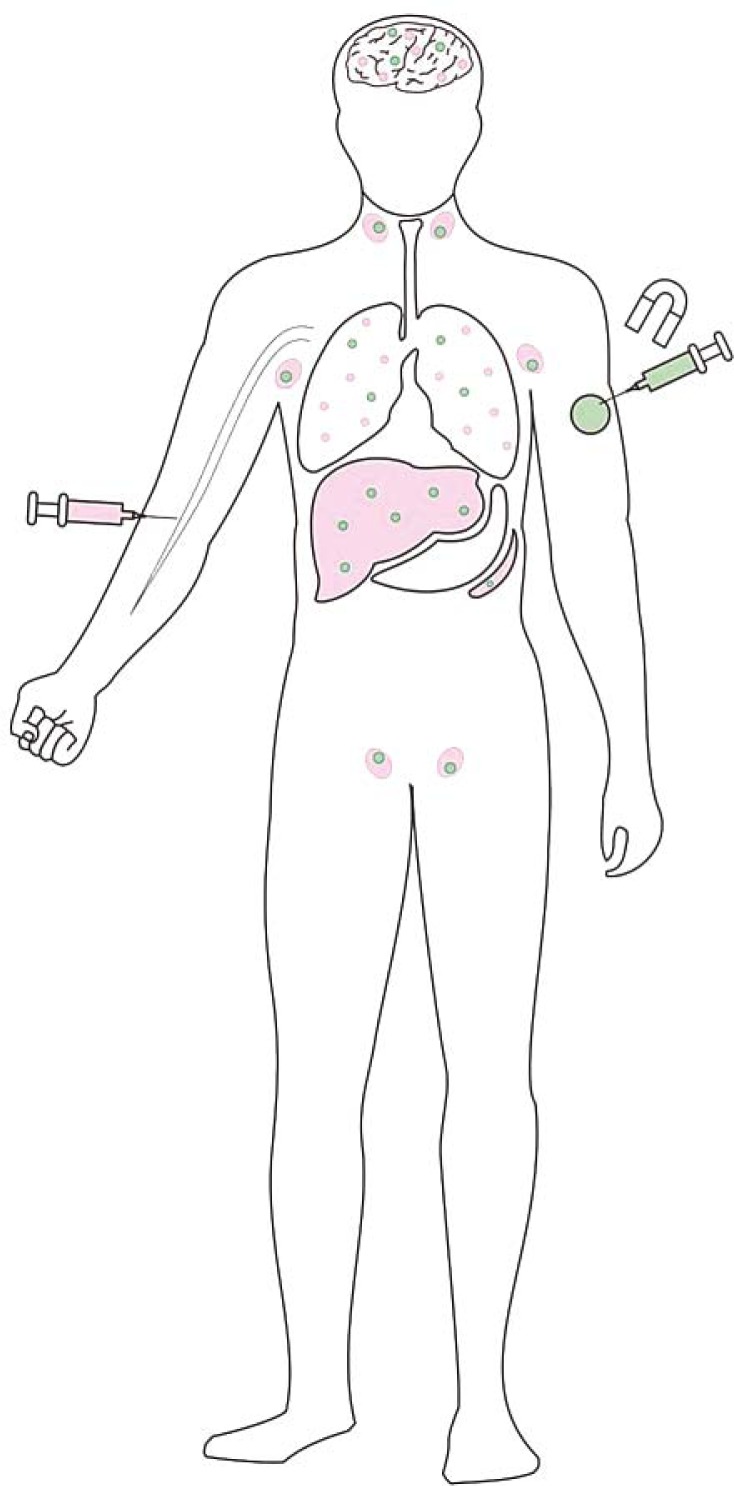
Biodistribution of magnetic nanoparticles within the body after different administration routes. After intravenous administration (pink syringe) magnetic nanoparticles predominantly accumulate in the liver, spleen and lymph nodes (pink areas). However, the blood flow also takes them to other organs, *e.g.* lungs, brain (pink dots). After intra-tumoral administration and exposure to the magnet (green syringe), magnetic nanoparticles concentrate in the tumor (green area). However, a small quantity can also be also found in the organs throughout the body, *e.g.* liver, lungs, lymph nodes, brain, spleen (green dots), which depends on leakage of the tumoral vasculature.

Different *in vivo* studies in mice and rats showed that magnetic nanoparticles after intravenous administration predominantly accumulated in the liver and spleen: 55% of the injected iron composing 190 nm magnetic nanoparticles localized in the liver after 6 h, but was reduced to 20% after 24 h.^57^ The level of iron in the spleen after 3 weeks corresponded to 25% of the injected dose.^58^ Significantly increased iron levels were also detected in the heart and brain, however these were notably lower than these in the liver and spleen.^57^ Biodistribution of magnetic nanoparticles in the mice after intra-peritoneal route was similar to that of intravenous administration: the highest concentrations of magnetic nanoparticles were observed in liver and spleen.^59^ If magnetic nanoparticles are guided by means of a magnetic field, they concentrate in the area of interest (Figure 3). For example, when 70 nm magnetic nanoparticles were injected into mice through the tail vein and directed to the heart and kidneys by means of magnetic field with flux density of only 2.5 mT for 6 h, they concentrated in the heart and kidneys as well as in the lungs.^26^ Biodistribution of magnetic nanoparticles after different administration routes is schematically presented in the Figure 3.

## Toxicity studies

### Toxicity of magnetic nanoparticles

Toxicity studies of magnetic nanoparticles are scarce. The first tolerance study with carbohydrate-coated magnetic nanoparticles as potential delivery systems was performed in nude mice and showed no median lethal dose (LD_50_), no alterations in the blood haematological and biochemical profiles as well as no organomegalies were observed after injection of magnetic nanoparticles. However, when 10–20% of the blood volume was infused with the ferrofluid, short episodes of lethargy and resistance of food uptake were detected.^25^ On the other hand tartrate and citrate-coated magnetic nanoparticles, administrated intra-peritoneally in mice, caused severe inflammatory reactions in the peritoneal cavity and around the hilum of spleen and kidneys, indicating that adsorption of carboxylic acids at physiological pH and isotonic conditions did not inevitably result in a biocompatible ferrofluid.^60^ Coating of magnetic nanoparticles with dextran despite of more than 6 months retention in the liver and spleen of mice caused no alterations observable in histology specimens of these organs.^61^ Similarly, histological analysis of liver, spleen and kidney after intravenous administration of oleic acid-coated magnetic nanoparticles did not show any alterations in these organs. However, lipid peroxidation indicating oxidative stress was elevated and returned to a normal value within 3 weeks.^57^

### Toxicity of an external magnetic field

Questions remain about the possibility of adverse side effects related to electromagnetic fields. According to the U.S. National Institute of Environmental Health Sciences (NIEHS), there is a weak association between magnetic field exposure of flux density as small as 0.0003 mT and an increased risk of childhood leukemia.^62^ According to numerous MRI examinations, a static magnetic field of flux densities from 0.5 to 2 T does not cause any known side effects and therefore the patient compliance is high.^63^ On the other hand, rats developed aversive and avoidance behavior when the field was increased up to ultra-high fields of flux densities of 4 T and 7 T, respectively.^64^

There are some inconsistent reports about cellular toxicity or adverse side effects caused by magnetic field exposure which might be due to the cell type dependent mechanisms. It seems that cells deriving from mesenchymal descent are more prone to the magnetic field exposure than other normal and malignant cells.^47^,^65^–^68^ Evidently, in order to provide assurance that the magnetic field accurately does not cause any side effects there is a vital need to perform additional *in vitro* as well as *in vivo* studies.

## Applications of magnetic nanoparticles in oncology

### Diagnostic purposes

For diagnostic purposes magnetic nanoparticles are utilized as contrast enhancing agents for MRI in order to improve spatial resolution and provide earlier lesion detection.^69^ SPIONs are replacing paramagnetic gadolinium-based MRI contrast agents due to their superior *in vivo* behavior and biocompatibility with some of them already being FDA-approved. Earliest magnetic nanoparticles for MRI were administered into the bloodstream and within minutes cleared by mononuclear phagocytic cells of the reticuloendothelial system. Their subsequent accumulation into the liver and spleen improved visualization of focal lesions with a few millimeters diameter.^70^–^72^ In 1996, the first liver-specific MRI contrast agent, Feridex I.V.^®^, came to the market which was soon followed by GastroMARK^®^, a contrast agent for MRI of the gastrointestinal tract. Modification of physicochemical properties of magnetic nanoparticles resulted in their prolonged blood half-life and vascular penetration which enabled visualization of other tissues and organs within the rats.^73^,^74^ For example, Chertok *et al.* visualized accumulation of magnetically-guided nanoparticles in experimentally-induced rat gliosarcomas after intravenous administration by MRI.^75^ Currently, magnetic nanoparticles are being investigated for visualization of lymph node metastases which are otherwise undetectable by existent technology equipment.^76^ Moreover, as reviewed by Jain *et al*., SPIONs with minor macrophage uptake and prolonged blood half-life have found preferential application in sentinel lymph node imaging as contrast enhancing agents for MRI.^77^

Concerning diagnostic, prognostic and even therapeutic implications, magnetic nanoparticles are also used in magnetic-activated cell sorting (MACS^®^) for magnetic separation of different tumor cells and cancer stem cells out of the bloodstream or tissue by the recognition of CD surface antigens.^78^–^81^ Briefly, magnetic nanoparticles coated with immunospecific agents tag target cells which are then separated from other biological entities by passing through an external magnetic field.

### Therapeutic purposes

For therapeutic purposes no magnetic nanoparticles have yet been approved for clinical use. However, the majority are being investigated as drug or gene delivery systems whereas to a considerably smaller extent they are being explored for the treatment of cancer by magnetic hyperthermia.

#### Magnetic hyperthermia

Magnetic hyperthermia is local therapeutic modality for the treatment of cancer which is founded on the fact that magnetic nanoparticles produce heat when exposed to an alternating current (AC) magnetic field. The therapy comprehends administration of magnetic nanoparticles into the tumor followed by an AC magnetic field exposure. Cancer cells loaded with magnetic nanoparticles are subjected to irreversible damage of temperatures above 42–43°C whereas normal cells withstand temperatures up to 46°C.^39^ Moreover, heat alters some receptor molecules on the surface of cancer cells which enhances their recognition by natural killer cells.^82^ In 2005, the first phase I clinical trial was carried out in a patient with a recurrent prostatic tumor concluding that magnetic hyperthermia is a feasible and well tolerated treatment modality.^83^ Two years later in combination with radiotherapy, magnetic hyperthermia was performed in 14 brain-cancer patients demonstrating that the therapy was well tolerated by all the patients with minimal or no clinical effect.^84^

### Drug carriers

Magnetically-guided drug carriers in the treatment of cancer date back to the late 1970s, however, no such magnetic nanoparticles have yet been clinically approved. Only nanoparticles without magnetic properties, *i.e.* liposomes encapsulating anthracyclines (daunorubicin and doxorubicin), and nanoparticulate albumin-bound paclitaxel are used for the treatment of different types of solid tumors and metastatic breast cancer, respectively.^85^

The idea of using magnetic microspheres as vehicles for drug delivery in cancer therapy was first introduced by Widder *et al*.^1^ In 1983 they performed the first preclinical study in rats. Selective targeting with intravenously administrated magnetic albumin microspheres containing low doses of doxorubicin resulted in total remission of 77% (17/22) of tumors after only one regimen of drug therapy.^86^

As late as 1996, the very first preclinical and clinical studies on magnetic nanoparticles for cancer therapy were done. It is essential to mention that the following magnetically-guided drug carriers were barely classified among nanoparticles since they measured 0.5 - 5.0 μm. A preclinical study of magnetically-guided nanoparticles as delivery systems in cancer therapy was performed in a xenotransplanted human colon as well as renal cancer tumor-bearing mice. After intravenous injection and magnetic guidance of epidoxorubicin attached to magnetic nanoparticles, complete remissions of tumors were observed.^25^ The first clinical phase-I magnetically-guided drug-targeted study was carried out in 14 patients with advanced unsuccessfully pretreated solid tumors. Intravenous administration of epidoxorubicin attached to magnetic nanoparticles resulted in transient serum iron elevations in almost all patients after the therapy, which did not cause any clinical symptoms, and in some patients increased ferritin levels in the blood were observed. In 50% (7/14) of the patients, magnetic nanoparticles were detected within the tumors. However, only a slight reduction of tumor size occurred in merely 14% (2/14) of the patients.^87^

Later, the same research group utilized mitoxantrone attached to magnetic nanoparticles of the ferrofluid (magnetic liquid) (FF-MTX) aiming to compare the antitumor efficacy of FF-MTX given by different administration routes. The treatment was performed in rabbits bearing squamous cell carcinomas (VX-2) which showed complete and permanent remission after intra-arterial administration of FF-MTX. However, intravenous application of FF-MTX did not result in statistically significant tumor remission in comparison to the control group.^88^

The following preclinical and clinical trials of two research groups focused on the delivery of doxorubicin hydrochloride adsorbed to magnetic targeted carriers (MTC-DOX) by selective arterial catheterization of the hepatic artery.^89^–^91^ A preclinical trial was performed in a swine model. By magnetic targeting, extravasation of MTC-DOX through the vascular wall was obtained, leading to their localization and retention in the tissue at the targeted site. The severity of liver necrosis correlated to the severity of embolization following treatment and was observed only in the animals which received the highest dose of MTC-DOX whereas no adverse effects were determined at the MTC-DOX low-dose group.^89^ Clinical trials with MTC-DOX were carried out in patients with inoperable hepatocellular carcinomas. No clinically significant toxicity was observed. However, all patients experienced abdominal pain during MTC-DOX administration which was intravenously controlled with analgesics.^90^,^91^ In the first phase I/II study, localization of MTC-DOX in the tumors was achieved in 94% (30/32) of all the patients with one complete and two partial responses.^90^ In the second study MTC-DOX was observed in 100% (4/4) of the tumors with 64–91% of the tumor volume loaded with magnetic nanoparticles. However, this resulted in only one partial response.^91^ A subsequent phase II/III multinational clinical study with MTC-DOX enrolling 240 patients with hepatocellular carcinoma was prematurely stopped as there was no increase in median survival time for MTC-DOX treated patients relative to patients treated with IV doxorubicin.^92^

To sum up, preclinical studies turned out in complete and permanent tumor remission; however, dose escalation clinical trials resulted in no clinically significant toxicities but had a relatively poor tumor response.

#### Nucleic acid carriers

In addition to chemotherapy, recent progress in gene therapy has made it a realistic option for the treatment of cancer.^93^,^94^ The idea of using magnetic nanoparticles as gene delivery systems emerged in the year 2000, combining expertise of chemistry, biology, medicine and physics. This new interdisciplinary approach has already shown some promising results in preclinical studies.^95^–^98^

Until the new millennium, the majority of studies focusing on gene delivery for therapeutic approaches used viruses as transport vehicles for nucleic acids. In order to avoid the disadvantages of viral based gene delivery, such as receptor dependent host tropism, pre-existing immunity of the host, induced immune response by the virus, potential recombination of viral and host cell genetic material and large-scale infrastructure for virion production, new methods have begun developing.^99^

Non-viral methods of gene transfer can be divided into three major groups: injection of naked plasmid DNA (pDNA), chemical and physical approaches.^100^ In 1990, first *in vivo* study injecting naked pDNA into mouse muscle was performed. In the injected tissue significant elevations of all three reporter genes encoding chloramphenicol acetyltransferase, luciferase and beta-galactosidase were observed.^101^ Later, injection of naked pDNA was repeated by others, as well as in other organs.^102^,^103^ In an effort to increase transfection efficiency, development of various physical approaches has begun. The general principle of nucleic acid internalization by physical approaches, which include microinjection, hydrodynamic delivery, biolistics, electroporation, sonoporation and impalefection, is based on disruption of the cell membrane to facilitate nucleic acid uptake.^104^–^109^ However, nucleic acids still remain exposed to biochemical degradation which reduces transfection efficacy; thus attaching or encapsulating nucleic acids within nanoparticles, which mediate their internalization by membrane fusion and/or endocytosis, results in increased transfection efficiency *in vitro* in comparison to some physical approaches.^110^

**FIGURE 4.**
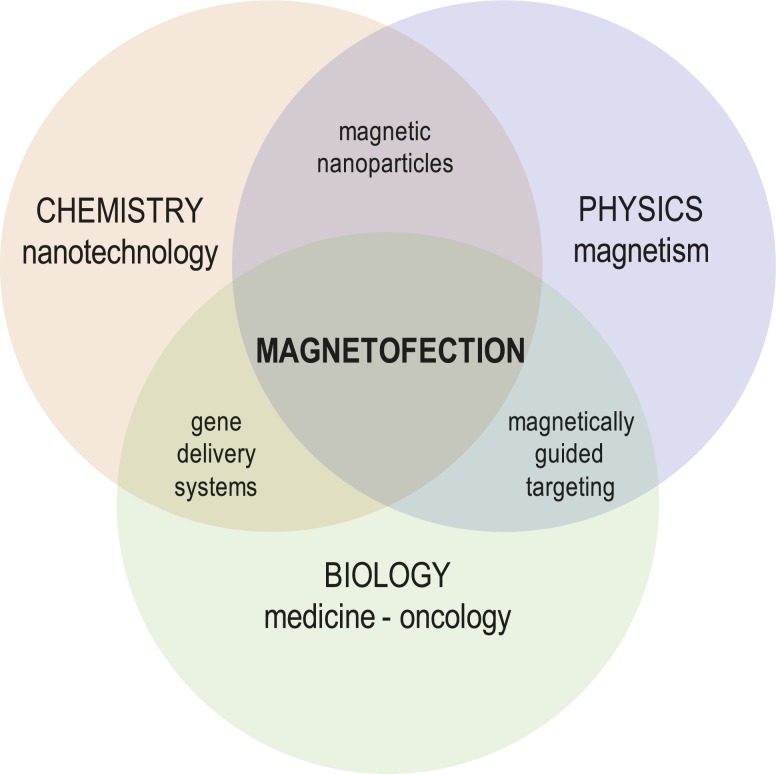
Schematic presentation of interdisciplinary approach resulting in magnetofection at the cross-section.

Progress in the field of nanotechnology and new trends in gene biology contributed to the development of a novel method called magnetofection, which unites the advantages of physical and chemical approaches in one system (Figure 4). The method is based on binding the nucleic acids to magnetic nanoparticles that concentrate and transfect cells in the area of interest by means of a magnetic field.^2^ For cancer therapy, a high-field, high-gradient, rare earth permanent magnet, such as Nd-Fe-B magnet, is placed above the solid tumor in order to retain administrated magnetic nanoparticles with bound nucleic acids *in situ* until they internalize and transfect malignant cells.^97^,^98^ Although magnetofection is considered a non-viral method of gene transfer, viral vectors can be supplementary attached to magnetic nanoparticles in order to additionally increase transfection efficiency.^111^ In 2000, the use of magnetic microparticles for transfection *in vitro* was first demonstrated in carcinoma C12S cells and *in vivo* in mice using an adeno-associated virus linked to magnetic microspheres *via* heparin. The study resulted in enhanced green fluorescent protein (GFP) expression due to the increase in contact between the delivery system and the cell.^112^ In these terms, the immunity-based problems arising from the use of viral vectors for gene transfer remain; therefore, studies of virus-associated magnetic nanoparticles will not be discussed in this review.

#### Magnetofection in vitro and ex vivo

Currently there are several commercially available magnetic nanoparticles measuring 50 to 200 nm in diameter, *e.g.* CombiMAG, PolyMAG and TransMAG^PEI^, which have been used in many *in vitro* and some *in vivo* studies of magnetofection.^2^,^28^,^31^,^42^,^95^–^98^,^113^ It is noteworthy that these vectors represent a hybrid system characterized by the iron oxide inner core and a coat consisting of PEI, which is a well known transfection agent.^114^

All *in vitro* studies confirmed efficient magnetofection of a variety of cell lines with various nucleic acids, in most cases pDNA followed by small interfering RNA (siRNA), short hairpin RNA (shR-NA) and antisense oligonucleotides, associated to magnetic nanoparticles and guided by a magnetic field generated by Nd-Fe-B magnets.^2^,^28^,^31^,^42^,^95^,^96^ Magnetofection of pDNA encoding GFP on mouse melanoma B16F1 cells is presented in Figure 4. The studies also demonstrated that magnetofection is superior to other standard transfection protocols, mostly lipofection.^95^,^115^

The first *in vitro* study demonstrated enhancement in *LacZ* reporter gene transfection of mouse embryonic fibroblasts NIH3T3 and Chinese hamster ovary (CHO) cells up to several 100-fold compared to transfection in the absence of magnetic field. In addition, a minimal dose of pDNA (0.1 μg) was sufficient to achieve high transfection levels.^2^ The highest increase in transfection efficiency of human umbilical vein endothelial cells (HUVEC) with the *luciferase* reporter gene by magnetofection was about 360-fold compared to various conventional transfection methods.^95^ However, it is worth to note that magnetic nanoparticles in the latter study were additionally coupled to lipid-based transfection reagents, Effectene^®^ and FuGENE^®^, as well as to a combination of the polymer-lipid transfection enhancer PEI/DOTAP-cholesterol, which greatly contributed to the increase in transfection efficiency. Other *in vitro* and *ex vivo* magnetofection-based studies also showed enhanced transfection efficacy of *luciferase*, *enhanced GFP* (*EGFP)* and *Discosoma sp. red fluorescent protein* (*DsRed)* reporter genes to various cell lines, however, to a lesser extent, *i.e.* from 3-fold to 36-fold.^116^–^119^

Refinements of the technique resulted in significantly reduced time needed for the transfection process to be completed (transfection time) in comparison to other non-viral gene delivery approaches. Under magnetic field guidance, the transfection time of HUVEC with oligodesoxy-nucleotides (ODN) against the p22^phox^ subunit of endothelial NAD(P)H-oxidase bound to magnetic nanoparticles was decreased to a few minutes whereas it required 24 h when ODN were coupled only to Effectene^®^.^96^ Cationic lipid-coated magnetic nanoparticles associated with transferrin demonstrated a 300-fold increase in transfection efficiency of the *luciferase* reporter gene in comparison to well established and efficient PEI polyplexes and Lipofectin^™^ after 15 min incubation.^115^ Similarly, Chorny *et al*. managed to efficiently transfect aortic smooth muscle cells A10 and bovine aortic endothelial cells (BAEC) with the use of polymer-coated magnetic nanoparticles attached to pDNA encoding EGFP just after 15 min of exposure to the magnetic field. The negligible transfection was observed in the absence of the magnetic field.^120^

Therefore, magnetofection is defined as enhanced delivery of nucleic acids associated with magnetic nanoparticles to the cells under the influence of a magnetic field.^2^

The majority of magnetofection studies utilized a static magnetic field, however, two research groups have also focused on the application of a pulsed magnetic field. The Swiss group utilized electromagnets and reported that transfection efficiency of reporter genes was the highest when magnetic nanoparticles were first sedimented by exposure to the permanent magnet before application of the pulsating magnetic field.^121^ The mechanism behind this observation could be an alteration in the permeability of cell membranes by pulsed magnetic field after the increased sedimentation by a static magnetic field enhanced the contact between the cells and magnetic nanoparticles. In another study of the same group, at least a 6-fold increase in transfection efficiency of the *EGFP* reporter gene to various primary cell lines was shown when a combination of static and pulsating magnetic field was used compared to the presence of static magnetic field alone. The transfection was the lowest when the cells were exposed only to the pulsed magnetic field.^119^ On the other hand, the American group utilized a computer-controlled stepper motor-driven horizontally oscillating magnet array system which produced increased magnetic field strength and a gradient with no heating in comparison to electromagnets used by the Swiss group. The lateral motion of the horizontally oscillating magnet array system at an amplitude 200 μm and frequency 2 Hz promoted extra energy and mechanical stimulation which increased particles sedimentation onto the cellular surface. The result was a 4-fold greater transfection of the *luciferase* reporter gene to human lung epithelial NCI-H292 cells than that of Lipofectamine^™^ 2000 and more than a 2-fold greater than transfection performed under a static magnetic field. The oscillating array system also had little or no effect on cell viability.^122^

#### Magnetofection in vivo

Although authors of several studies have reported about the suitability of magnetofection of reporter genes *in vitro*, some improvements are still required to make the method efficient enough to be widely used for *in vivo* applications.

To demonstrate that magnetofection of reporter genes *in vivo* is feasible, two studies using pDNA encoding beta-galactosidase and luciferase were performed in rats, mice and pigs.^2^,^113^ In addition, a magnetofection study using Cy3-fluorescence-labeled antisense ODN was carried out in mice by the same extended research group.^96^ Initial pre-clinical *in vivo* trial of *LacZ* reporter gene delivery was performed in ilea lumens of rats using viral vector-free magnetic nanoparticles and in stomach lumens of mice using adenovirus-associated magnetic nanoparticles. Efficient transfection was observed in lamina propria of ileum as well as in crypts of fundic glands after 20 min of exposure to the magnetic field. For additional proof-of-principle, magnetofection of the *luciferase* reporter gene was done in the ear veins of pigs. Luciferase expression was observed in all vein samples under the influence of magnetic field whereas no transfection was found distally from the magnet position and in other organs.^2^ In another study, the same magnetic nanoparticles were coupled to lipid 67 (GL67) and pDNA encoding luciferase, thus forming ternary complexes. GL67 is a cationic lipid considered as the gold standard for *in vivo* airway gene transfer. The authors aimed to compare magnetofection efficacy of ternary complexes to transfection efficacy of plane GL67/pDNA. Surprisingly, *in vivo* transfection of the murine nasal epithelium with plane GL67/pDNA resulted in an approximately 90-fold higher luciferase expression than that observed by magnetofection. The authors referred the poor outcome of magnetofection to the size of magnetic nanoparticles (200 nm), their coating and characteristics of the magnetic field applied, suggesting that smaller particles with a modified surface coating could have been more efficient in crossing extracellular barriers as well as intracellular membranes in the airway epithelium.^113^ The third *in vivo* study was performed in mice in order to investigate whether magnetofection is feasible strategy for directing antisense ODN, complexed to magnetic nanoparticles, to a targeted site *via* arterial catheterization. Nd-Fe-B magnet was held above the right testis throughout the infusion of Cy3-labeled antisense ODN, coupled to magnetic nanoparticles, *via* femoral catheter and for another additional 4 min. The study demonstrated site-specific magnetofection of the ipsilateral arterioles of the cremaster muscle whereas contralateral vessels of the same muscle, which were not exposed to an external magnetic field, were not transfected.^96^

Magnetofection of therapeutic genes *in vivo* was published in two papers, both dealing with veterinary clinical trial consisting of dose-escalation neoadjuvant gene therapy to surgery.^97^,^98^ The aim of neoadjuvant immunostimulatory gene therapy in the treatment of cancer is to induce local production of cytokines which triggers systemic anti-tumor immunity.^123^ Both studies were carried out in feline fibrosarcomas by the same research group in which immunostimulatory therapeutic genes were applied by magnetofection. In the study of Jahnke *et al.*, dose-escalation study was performed with a combination of pDNA encoding three different cytokines: feline interleukin-2 (feIL-2), feline interferon-gamma (feIFN-γ) and feline granulocyte-macrophage colony-stimulating factor (feGM-CSF). Altogether six cats developed local recurrences during 1-year observation period, four of them received the highest dose of the total amount of pDNA (1350 μg pDNA; 450 μg per plasmid).^97^ That was clarified by bell-shaped dose dependence of IL-2.^124^,^125^ Moreover, one cat in this group (12.5%) showed adverse events. However, the study concluded that the highest dose was well tolerated as the only adverse events occurred once and were self-limiting. Due to the early recurrences the authors suggested to include in a subsequent phase-II study also the second highest dose of pDNA (450 μg pDNA; 150 μg per plasmid).^97^ In the study of Hüttinger *et al.*, magnetofection of pDNA encoding feGM-CSF was performed 14 days prior to surgery. Results of the study demonstrated that ten of the treated animals (50%) were recurrence-free after 360 days of observation. Moreover, the highest dose (1250 μg) of pDNA applied was shown to be safe for phase-II testing.^98^

## Future directions

New technologies have enabled synthesis of biocompatible magnetic nanoparticles that can be functionalized with therapeutic molecules. The transfection method using magnetic nanoparticles, which are manipulated by an external magnetic field, is called magnetofection. It is a promising strategy that can lead to targeted delivery of pDNA carrying therapeutic genes, siRNA or other gene therapy approaches. Due to its physical properties of delivery, the approach is feasible on different tissues; foreseen can be tumors, muscle, skin and others. In this field of research very little was done. When the protocols for synthesis of magnetic nanoparticles and their functionalization with nucleic acids are standardized along with contemporary optimization of magnetic field parameters, the field will open for broader and in depth investigations, that may in near future also bring magnetofection into the clinical trials.

## Figures and Tables

**FIGURE 5.**
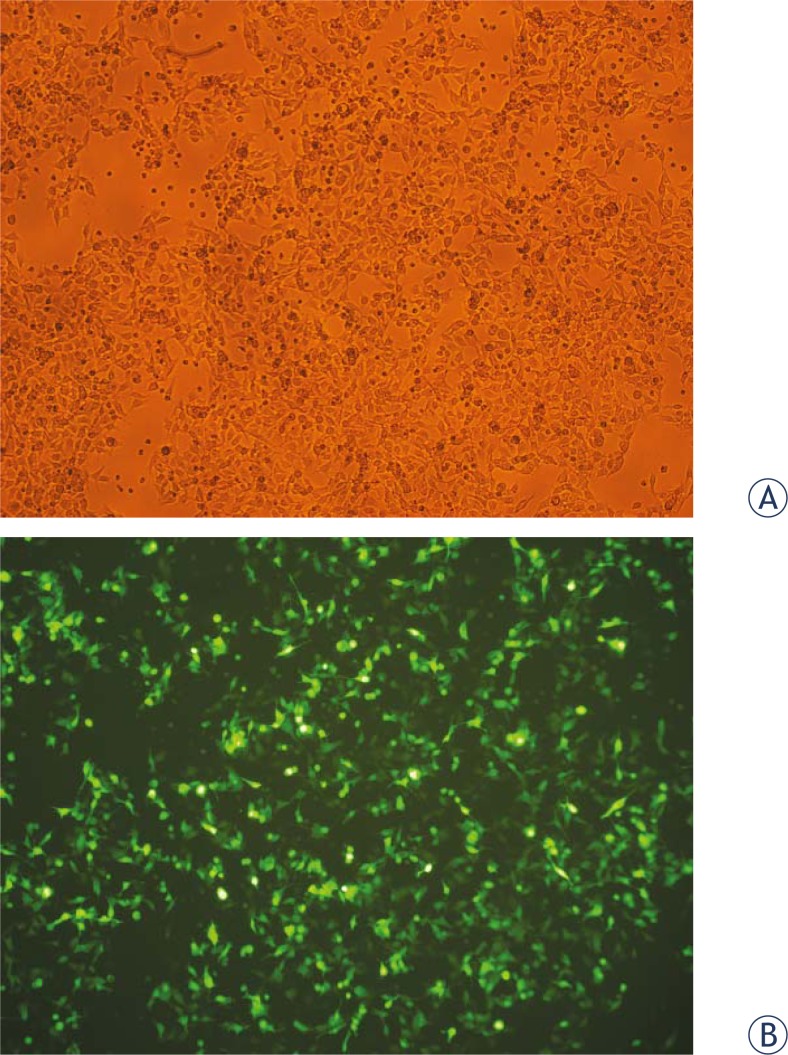
Photomicrograph of mouse melanoma B16F1 cells, taken 24 h after magnetofection of pDNA encoding GFP was performed, demonstrating high transfection efficiency. Image taken under visible light condition (A) and image taken under fluorescence epi-illumination (B) (x 60 magnification).

**TABLE 1.** Classification of nano-sized delivery systems by chemical compounds and shape. Magnetic nanoparticles which are most often used in biomedical applications are shadowed

**CHEMICAL COMPOUNDS**				**SHAPE**
ORGANIC	NATURAL	LIPIDS	Egg phosphatidylcholine (EPC), egg phosphatidyl glycerol (EPG)	Liposomes
		PROTEINS	Human serum albumin (HSA), gelatin	Nanoparticles*
		CARBON HYDRATES	Chitosan, alginate	Nanoparticles*
	SYNTHETIC	LIPIDS	Dipalmitoyl phosphatidylcholine (DPPC), dimyristoyl phosphatidylcholine (DMPC), dimyristoyl phosphatidylglycerol (DMPG), dipalmitoyl phosphatidic acid (DPPA), distearoyl phosphatidylcholine, cholesterol (Ch)	Liposomes
			Tricaprin, trilaurin, trimylistin, tripalmitin with gliceryl monostearate, cetyl palmitate, stearic acid	Solid lipid nanoparticles
		POLYMERS	**Homopolymers**: Poly(alkylcyanoacrylate) (PACA), poly(2-hydroxyethyl methacrylate) (pHEMA), poly(N-(2-hydroxypropyl)methacrylamide (pHPMA), polyvinylpyrrolidone (PVP), poly(methyl methacrylate) (PMMA), polyorthoesters, polycaprolactone (PCL), poly(vinyl alcohol) (PVA), poly(acrylic acid) (PAA), polylactides (PLA) **Copolymers**: Poly(alkylcyanoacrylate)-*co*-poly(ethylene glycol), poly(lactid acid)-*co*-poly(glycolic acid) (PLGA), poly(L,L-lactide-*co*-L-aspartic acid), poly(ethylene-*co*-vinyl acetate) (PEVA)	Dendrimers Nanoparicles* Nanocomposites Nanobrushes Nanotubes Micelles Nanogels
		SURFACTANTS	**Cationic**: Sodium dodecyl sulfate (SDS) **Anionic**: Cetyl trimethylammonium bromide (CTAB) **Non-ionic**: Copolymers of poly(ethylene oxide) and poly(propylene oxide)	Micelles
ORGANIC & INORGANIC		LIPIDS	DPPC/Ch/γ-Fe_2_O_3_, Fe_3_O_4_	Magnetic liposomes
	MAGNETIC	POLYMERS	Ni-Zn-ferrite/SiO_2_, Fe-Ni/polymer, Co/polymer, PMMA/α-Fe_2_O_3_	Nanocomposites
INORGANIC		COMPOUNDS	Ni-Fe/SiO_2_, Co/SiO_2_, Fe-Co/SiO_2_, Fe/Ni-ferrite, Ni-Zn-ferrite/SiO_2_	Nanocomposites
		COMPOUNDS	**Iron**: **γ-Fe_2_O_3_**, **Fe_3_O_4_**	Nanoparticles*
			MgFe_2_O_4_, MnFe_2_O_4_, FePt, NiFe_2_O_4_	Nanoparticles* Nanorods
			**Nickel**: NiO, NiFe_2_O_4_	
			**Cobalt**: Co_3_O_4_, CoFe_2_O_4_	
			**Manganese**: Mn_3_O_4_, MnO_2_	
	NON-MAGNETIC		CdSe/ZnS	Nanocrystals
			ZnO, Au, Ag, Cu, CdSe/ZnS, GaN, TiO_2_, C, TiC, VO_2_, V_2_O_5_, PbS, CdS, SiC, BiPO_4_, AOB	Nanorods Nanoparticles*
			Calcium phosphate	Nanocomposites
		ELEMENTS	C	Fullerenes Nanotubes

* Nanoparticles include nanocapsules and/or nanospheres
